# Cigar Use Misreporting Among Youth: Data from the 2009 Youth Tobacco Survey, Virginia

**Published:** 2012-01-19

**Authors:** Aashir Nasim, Melissa D. Blank, Brittany M. Berry, Thomas Eissenberg

**Affiliations:** Virginia Commonwealth University, Department of Psychology; Virginia Commonwealth University, Department of Psychology, Richmond, Virginia; Virginia Commonwealth University, Department of Psychology, Richmond, Virginia; Virginia Commonwealth University, Department of Psychology, Richmond, Virginia

## Abstract

**Introduction:**

Researchers have suggested that adolescents' cigar use has increased beyond the rates being reported on tobacco use surveys. Differences in content knowledge and everyday colloquial expressions may be responsible for misreporting of cigar use. To determine whether cigar use is subject to systematic misreporting, we compared reports of general cigar use ("During the past 30 days, on how many days did you smoke cigars, little cigars, and cigarillos?") with reports of brand-specific use ("During the past 30 days, on how many days did you smoke Black & Milds?") among a statewide sample of adolescents in Virginia.

**Methods:**

We examined data from 3,093 youth who completed the 2009 Virginia Youth Tobacco Survey to determine differences in the rate of misreported cigar use (ie, those who reported Black & Mild use but did not report cigar, little cigar, or cigarillo use) for youth with varying demographic profiles and conditions.

**Results:**

More than one-half of Black & Mild users (57.3%) did not report general cigar use. Cigar use misreporting was most prevalent among older adolescents, blacks/African Americans, current users of cigarettes and hookah, and youth diagnosed with asthma.

**Conclusion:**

General cigar-use items on statewide surveys significantly underestimate the prevalence of youth cigar use. More comprehensive measures of cigar use may be beneficial in assessing tobacco use among groups most likely to misreport their tobacco use, such as African Americans and youth diagnosed with asthma.

## Introduction

After cigarettes, cigars are the most widely used tobacco product among adolescents aged 12 to 17, and national data show that 8.9% of adolescents currently use cigarettes, compared with 4% who report current cigar use (1). These rates of cigarette and cigar use are considerably lower than rates from 10 years ago. Since 2002, for example, tobacco use prevalence among youth has trended downward; cigarette and cigar use have decreased by 31% and 11%, respectively. Such appreciable declines in adolescent tobacco use are encouraging signs for public health and prevention.

Although tobacco use prevalence among US youth has declined (1-3), recent observations suggest that cigar use has increased during this same time period. Several reports describe an exponential increase in the sales of cigar products like little cigars and cigarillos (4-7). Moreover, recent tobacco industry marketing strategies to replace cigarettes with cigars (8,9), price inequities rendering cigars more affordable than cigarettes (10), and the absence of primary prevention programs that target cigar smoking (11), when taken together, suggest that critical issues exist in the surveillance of cigar use prevalence among youth populations.

One explanation for disparate cigar trend data is related to the measurement of cigar use behaviors. Tobacco surveillance systems employ survey measures that contain face validity (content that gives the appearance of measuring a construct) but may lack sufficient content validity (content included in the measure that is actually representative of the construct). Tobacco surveys, in general, use a standard cigar item ("During the past 30 days, on how many days did you smoke a cigar, little cigar, or cigarillo?") that on the surface appears to measure comprehensive cigar use. Yet, this standard item does not adequately describe what a cigar is and also assumes knowledge of all types of cigars (eg, *cigarillo*). Researchers have explained that the tobacco lexicon of public health officials likely differs from that of adolescents (8), and such differences in content knowledge and everyday colloquial expressions may lead to misreporting of cigar use (12). For instance, Yerger et al found that African American youth were more likely to report having ever smoked a cigar after a focus group discussion that clarified what was meant by the word *cigar* (12).

Cigar use misreporting is defined here as a discrepancy in the reporting of general (ie, cigars, little cigars, and cigarillos) and brand-specific (ie, Black & Mild) cigar use. Cigar use misreporting differs from tobacco underreporting, whereby youth fail to report lifetime or current use of a tobacco product. Moreover, misreporting of cigar use is detected by comparing responses to content-similar items on surveys, whereas underreporting of tobacco use is generally revealed through corroborating self-report measures of recent tobacco use and biochemical verification analyses (eg, saliva cotinine).

Although many studies have described underreporting (13), only 1 systematic investigation has reported on possible misreporting related to cigar use (14). In that study, researchers collected tobacco use data in 2002 and 2004 from high school students in a Midwest US county. Cigar use was measured with an aggregate item in 2002, and in 2004, this item was modified to measure brand-specific use (ie, Black & Mild). Their findings show that the percentage of students reporting cigar use rose from 12.9% in 2002 to 20.7% in 2004, indicating that item modification led to greater detection of cigar use in the subsequent year. Although this study does not eliminate alternative explanations for increased cigar rates (eg, an actual increase in cigar use from 2002 to 2004), results that show a dramatic rise in cigar use when brand-specific responses are presented do support the hypothesis that cigar use is being misreported.

To address the idea that cigar use is subject to systematic misreporting, we examined cigar use responses from a statewide sample of adolescents in Virginia. We also sought to identify demographic characteristics of adolescents who are most likely to misreport cigar use.

## Methods

A secondary data analysis was conducted by using responses from the 2009 Virginia Youth Tobacco Survey (YTS), an ongoing, statewide monitoring and surveillance survey of tobacco use conducted by the Virginia Tobacco Settlement Foundation (VTSF), Centers for Disease Control and Prevention (CDC), and the Survey and Evaluation Research Laboratory (SERL) at Virginia Commonwealth University (VCU). This study draws from a representative sample of middle and high school students who completed the YTS in 2009 (N = 3,928). A total of 48 middle schools and 50 high schools were randomly selected to participate. Of those, 34 middle schools (70%) and 36 high schools (72%) agreed to participate. A total of 2,368 students were eligible to participate in selected classrooms at middle schools, and 2,232 students were eligible in selected classrooms at high schools. Approximately 89% of those eligible in middle school classrooms (n = 2,101) and 82% of those eligible in high school classrooms (n = 1,827) returned useable surveys (total N = 3,928). A full description of the research design and the 2-stage sampling procedures has been published elsewhere (http://www.healthyyouthva.org/vtsf/data/youth-tobacco-survey.asp) (15-17).

### Measures

Respondents reported current use of cigarettes, smokeless tobacco (SLT), and waterpipe (eg, hookah, shisha, nargile). In addition, respondents provided information on general and brand-specific cigar use: "During the past 30 days, on how many days did you smoke cigars, cigarillos, or little cigars?" and "During the past 30 days, on how many days did you smoke Black & Milds?" All tobacco use items were recoded as dichotomous variables indicating past 30-day use (0 = none/did not smoke in the past 30 days; 1 = smoked 1 or more days in the past 30 days). Two additional tobacco use variables were created by using cross-tab frequencies for general (0, 1) and brand-specific (0, 1) cigar use items. Adjusted cigar use was coded as follows: 1 = [general (1) and brand-specific (0)] or [general (0) and brand-specific (1)] and 0 = [general (0) and brand-specific (0)]. Misreported cigar use was coded as 1 = [general (0) and brand-specific (1)] and 0 = [all other frequency combinations].

Age (≤12 y Reference), sex (female Reference), race/ethnicity (non-Hispanic white Reference), discretionary income (<$1 Reference), and school type (middle school Reference) were examined. All items available on the 2009 YTS that describe the characteristics of those diagnosed with asthma were also included. Respondents reported if they had ever been diagnosed with asthma ("Has a doctor or nurse ever told you that you have asthma?"), incidence ("During the past 12 months, have you had an episode of asthma or an asthma attack?"), and severity ("During the past 12 months, about how many times did you visit an emergency room or urgent care center because of asthma?").

### Data analysis

Weighted data from the 2009 YTS (most recent year) were analyzed for this study. The data were weighted by CDC to account for unequal chances of selection, differential nonresponse rates, and demographics to include race, sex, and grade. Of the 3,928 participants surveyed, 3,093 reported complete information for items pertinent this study. Multiple imputation was used to correct for bias due to participant nonresponses and to ensure that data most accurately reflect youth populations in Virginia. Tests for proportions between unadjusted and adjusted cigar use percentages were computed. Logistic regression analyses were computed with Stata version 11 (StataCorp LP, College Station, Texas) to determine the likelihood of cigar use misreporting by youth sample characteristics.

## Results

The majority of the 2009 YTS sample was female (51.5%), white (57.3%), and between the ages of 13 and 16 (56.8%) (Table 1). Most respondents reported current use of cigarettes (9.2%) and Black & Milds (9.2%), followed by use of cigars (6.1%), waterpipes (4.9%), and smokeless tobacco (3.5%). Of the total sample, 23.7% reported being diagnosed with asthma, and 10.2% indicated having an asthma episode in the past year.

More than half of Black & Mild users (57.3%; n = 284) did not report current cigar use in 2009. This resulted in a significant increase in the rate of cigar use from 6.1% (unadjusted) to 11.4% (adjusted) in 2009 (*z* = 7.28, *P* < .001) (Figure). The results of the univariate analyses show that older adolescents were more likely than those aged 12 or younger to misreport cigar use (odds ratios [ORs] ranged from 2.13 to 3.28) (Table 2). Black/African American adolescents were more likely than white/European American youth to misreport use. Cigar use was also misreported for current users of cigarettes and waterpipes. Among subpopulations, misreported cigar use was highest among youth diagnosed with asthma (OR, 1.80; 95% CI, 1.19-2.73) and among those with the greatest asthma severity (OR, 2.13; 95% CI, 1.39-3.25) (data not shown).

**Figure.**
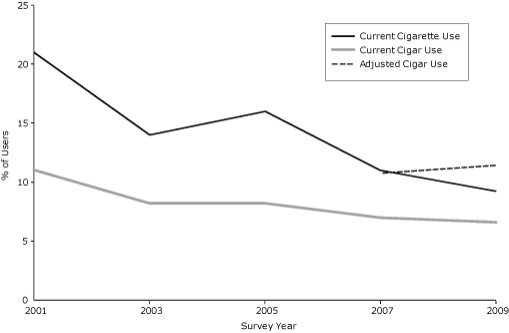
Trends in current cigarette, cigar, and adjusted cigar use among youth, Virginia, 2001-2009. An item assessing brand-specific cigar use was added to the Virginia Youth Tobacco Survey in 2007 and 2009. Adjusted cigar use is based on 2007 and 2009 data corrected for responses to brand-specific items versus general items. Tests for proportions were computed to determine differences between current and adjusted cigar use percentages.

**Table:** 

	**2001**	**2003**	**2005**	**2007**	**2009**
**Current Cigarette Use**	21	14	16	11	9.25
**Current Cigar Use**	11	8.2	8.2	7	6.6
**Adjusted Cigar Use**			8.2	10.75	11.4
**N**	9,400	2,070	2,074	2,067	3,093

## Discussion

On the basis of this study and another (14), youth cigar use misreporting yields biased estimates of tobacco use prevalence. Specifically, cigar prevalence rates may not be reliable due to the misreporting of information on currently available tobacco use surveillance systems. Given the manner in which cigar products are defined on these measures, cigar use may be inaccurately reported by some adolescent populations (12,14). Thus, we compared differences in the reporting of cigar products assessed via generic versus brand-specific questions.

Results showed that almost 60% of respondents who reported current use of Black & Milds (ie, cigarillo) did not report current use of "cigars, cigarillos, or little cigars." Accordingly, the rate of cigar use among this sample nearly doubled when rates were adjusted for this subgroup of respondents. These results are similar to those of the only other available study on the misreporting of cigar use; prevalence rates increased by approximately 8% when a general item (ie, past 30-day use of a "cigar, little cigar, or cigarillo") was modified to provide brand information (ie, past 30-day use of a "cigar, little cigar, or cigarillo [such as Black & Milds]")(14). Notably, this study used a successive independent samples study design and, thus, comparisons were made across assessment points (2002 vs 2004) and populations (n = 2,035 vs 1,537). Results may be a product, therefore, of an actual increase in the prevalence of cigar use during this 2-year period. The results reported here, however, are based on data collected from a single sample using the same questionnaire.

Another goal was to identify demographic characteristics of adolescents that may predict the misreporting of cigar use. Results revealed that misreporting was 2 to 3 times more likely among older respondents (5.95-11.5%) than those aged 12 or younger (2.9%), as well as 2 to 3 times more likely among black/African American adolescents (10.9%) than white/European adolescents (3.9%). Terchek et al (14) reported no differences in cigar use misreporting as a function of age but higher rates for black respondents (10.3%) than for white respondents (6.8%). Considered collectively, these findings suggest developmental and cultural factors may play a role in the interpretation of cigar use items on tobacco use surveillance measures. Thus, the validity of some standard tobacco use items may be  questionable on the basis of disparate response patterns across racial/ethnic groups.

This study also contributes to the growing literature on tobacco use among youth diagnosed with asthma. Previous studies show that youth diagnosed with asthma smoke cigarettes at 1.5 times the rate of otherwise healthy youth (18). In this study, 30.5% of youth diagnosed with asthma reported past 30-day cigar use (adjusted) compared with only 11.1% of the entire youth sample. Moreover, two-thirds (68.2%) of these youth smokers with asthma misreported cigar use; that is, most reported use of Black & Milds but not "cigars, little cigars, or cigarillos."

The finding that youth diagnosed with asthma are more likely than the general population to smoke cigars and to misreport cigar use raises questions. Little is known about how these youth reconcile their current medical diagnosis with engaging in behaviors that seem to exacerbate their condition (eg, smoking tobacco). Given perceptions among sampled youth that cigars are less harmful than cigarettes (19-21), future work might examine perceptions about the influence of cigar versus cigarette smoking on asthmatic symptoms among this population. For example, these youth may perceive that cigar products contain fewer toxic ingredients than cigarettes (22) or are harmful only when the cigar smoke is inhaled (23-24). The influence of peers is another consideration. Youth diagnosed with asthma may have increased vulnerability to peer pressure and substance use behaviors like smoking (25). Peers may help to shape youths' perceptions that Black & Mild use, for example, differs from cigarette and cigar use both in terms of content (ie, smoke toxicants) and smoking style or behavior (ie, the extent to which smoke is inhaled). Research on this acute medical subpopulation may benefit from exploratory studies that attempt to further elucidate perceptions of cigar use.

Our study has limitations. The generalizability of this study is limited to youth enrolled in middle and high schools in Virginia. Many tobacco surveillance systems have not incorporated brand-specific cigar use items or product descriptions in their surveys. Thus, we are unable to conclude whether cigar misreporting is a robust finding or simply a measurement artifact. In addition, the influence of logic errors recorded in surveys when youth report inconsistent tobacco use behaviors (eg, a no response to lifetime smoking and a yes response to past-30 day cigarette smoking) must be considered. Although we are unable to determine if our findings are subject to logic errors, by way of comparison, the percentage of logic errors reported in the 2009 YTS related to cigarette use was less than 1%.

This study is among the first to address the view that cigar use items on statewide surveys contain face validity but may lack sufficient content validity. Estimated tobacco use prevalence rates have a substantial influence on federal and state tobacco control policy, resource allocation and priority funding for tobacco research, and the dissemination of tobacco use prevention curricula and materials. Researchers should consider developing comprehensive assessment strategies to better detect and monitor cigar use in youth populations, especially among African Americans and youth diagnosed with asthma. In addition, health professionals should consider incorporating more detailed tobacco use screening items to ascertain accurate information.

## Acknowledgments

Support for the first author was provided by the National Center on Minority Health and Health Disparities (NCMHD) and the VTSF no. 135002.

## Figures and Tables

**Table 1.** Demographic Characteristics for the 2009 Youth Tobacco Survey (YTS) Sample, Virginia

**Sample Characteristics**	N	Mean % (95% CI)

Total population	3,093	
**Age, y**		
≤12	920	29.7 (28.2-31.4)
13-14	996	32.2 (30.5-33.8)
15-16	761	24.6 (23.0-26.1)
≥17	416	13.5 (12.1-14.5)
**Sex**		
Male	1,501	48.5 (46.9-50.4)
Female	1,592	51.5 (49.5-53.1)
**Race/ethnicity**		
White/European American	1,772	57.3 (55.4-58.9)
Black/African American	739	23.9 (22.2-25.2)
Hispanic/Latino	193	6.2 (5.4-7.1)
Asian American	140	4.5 (3.7-5.2)
Multiple race/other	249	8.1 (7.2-9.1)
**School level**		
Middle school	1,564	50.5 (48.9-52.4)
High school	1,529	49.5 (47.5-51.0)
**Discretionary income per wk, $**		
<1	554	17.9 (16.6-19.3)
1-5	260	8.4 (7.4-9.4)
6-10	286	9.2 (8.3-10.3)
11-20	560	18.1 (16.6-19.3)
>20	1,433	46.3 (44.0-47.9)
**Current tobacco use **		
Cigarettes	286	9.2 (8.2-10.2)
Cigars	190	6.1 (5.2-7.1)
Black & Mild	284	9.2 (8.2-10.2)
Smokeless tobacco	110	3.5 (2.9-4.2)
Waterpipe	154	4.9 (4.0-6.0)
**Other youth characteristics**		
Diagnosed with asthma	735	23.7 (22.4-25.5)
Episode of asthma (past 12 mos)	314	10.2 (9.1-11.2)
**Emergency department visits (past 12 mos)**		
None	2,908	94.0 (93.2-94.9)
1-3	145	4.6 (3.9-5.4)
4-12	18	<1.0 (0-3.0)
>12 times	22	<1.0 (0-1.0)

Abbreviation: CI, confidence interval.

**Table 2.** Demographic Characteristics and Logistic Regression Results for Cigar Use Misreporting Among Youth, 2009 Youth Tobacco Survey, Virginia

**Sample Characteristics**	Odds Ratio (95% CI)
**Age, y**	
≤12	1 [Reference]
13-14	2.13 (1.16-3.90)
15-16	3.06 (1.40-6.65)
≥17	3.28 (1.42-7.58)
**Sex**	
Male	1 [Reference]
Female	0.87 (0.60-1.27)
**Race/ethnicity**	
White/European American	1 [Reference]
Black/African American	3.24 (2.16-4.88)
Hispanic/Latino	0.69 (0.26-1.80)
Asian American	1.03 (0.36-2.90)
Multiple race/other	0.98 (0.42-2.28)
**School level**	
Middle school	1 [Reference]
High school	0.68 (0.38-1.20)
**Weekly discretionary income per wk, $**	
<1	1 [Reference]
1-5	0.32 (0.9-1.07)
6-10	0.97 (0.37-2.56)
11-20	1.73 (0.83-3.60)
>20	1.88 (0.95-3.70)
**Current tobacco use**	
No past 30-d use	1 [Reference]
Cigarettes	5.38 (0.38-1.20)
Smokeless tobacco	0.94 (0.36-2.41)
waterpipe	3.69 (1.99-6.83)
**Other youth characteristics**	
Not diagnosed with asthma	1 [Reference]
Diagnosed with asthma	1.80 (1.19-2.73)
Episode of asthma (past 12 mos)	1.68 (0.88-3.21)
Emergency department visits (past 12 mos)	2.13 (1.39-3.25)

Abbreviation: CI, confidence interval.
